# The Effect of Interocular Phase Difference on Perceived Contrast

**DOI:** 10.1371/journal.pone.0034696

**Published:** 2012-04-02

**Authors:** Daniel H. Baker, Stuart A. Wallis, Mark A. Georgeson, Tim S. Meese

**Affiliations:** School of Life and Health Sciences, Aston University, Birmingham, United Kingdom; The University of Sydney, United States of America

## Abstract

Binocular vision is traditionally treated as two processes: the fusion of similar images, and the interocular suppression of dissimilar images (e.g. binocular rivalry). Recent work has demonstrated that interocular suppression is phase-insensitive, whereas binocular summation occurs only when stimuli are in phase. But how do these processes affect our perception of binocular contrast? We measured perceived contrast using a matching paradigm for a wide range of interocular phase offsets (0–180°) and matching contrasts (2–32%). Our results revealed a complex interaction between contrast and interocular phase. At low contrasts, perceived contrast reduced monotonically with increasing phase offset, by up to a factor of 1.6. At higher contrasts the pattern was non-monotonic: perceived contrast was veridical for in-phase and antiphase conditions, and monocular presentation, but increased a little at intermediate phase angles. These findings challenge a recent model in which contrast perception is phase-invariant. The results were predicted by a binocular contrast gain control model. The model involves monocular gain controls with interocular suppression from positive and negative phase channels, followed by summation across eyes and then across space. Importantly, this model—applied to conditions with vertical disparity—has only a single (zero) disparity channel and embodies both fusion and suppression processes within a single framework.

## Introduction

When presented with similar images to each eye, the human visual system combines them into a single percept. Yet dissimilar images, such as a bright region in one eye and a dim region in the other, are not fused in this way. Instead, they mutually suppress each other, often undergoing binocular rivalry (e.g. [1]). Although binocular fusion depends on image similarity (or interocular phase), a recent study on dichoptic masking concluded that suppression occurs for both similar and dissimilar images [2]. How do these two processes of fusion and suppression affect the perceived contrast of a binocular stimulus, and the spatial layout of the cyclopean (i.e. binocular) image?

In a key study by Ding and Sperling [3], [4], observers indicated the perceived location of the dark bar of a sine-wave grating, which was presented as two monocular component gratings of different spatial phases. When the monocular gratings had equal contrast, perceived location was determined by the average of the sine-waves shown to each eye. For unequal contrasts, the position was shifted towards the higher contrast component. The full pattern of results was explained by a binocular gain control model featuring suppression between the eyes, followed by binocular summation. But how is the perceived *contrast* of such a stimulus affected by the phases of the monocular stimuli that comprise it?

Ding & Sperling's model (and those like it) implicitly predicts that the perceived contrast of binocular stimuli will depend on interocular phase difference (since the model contains phase terms), but they did not explore these predictions, nor test them empirically. To investigate this, Huang et al. [5] carried out a binocular contrast matching experiment in which interocular phase difference was manipulated. Contrary to the model's predictions, they found no change in perceived contrast as a function of phase for phase differences up to 90° at fairly high contrasts (16, 32 & 64%). On the basis of these results, they constructed a ‘multi-channel’ computational model in which phase difference does *not* affect perceived contrast, but does affect perceived position.

We suggest that this conclusion may not be a general one, because the range of phase differences examined (0, 45 & 90°) and the high contrasts used in the Huang et al. study [5] comprise only a small subset of the available stimulus space. There is good reason to believe that at low contrasts (close to detection threshold), where binocular summation occurs for in-phase, but not antiphase (i.e. 180° phase difference) stimuli [6], [7], extreme phase differences could affect perceived contrast. However, this would have been missed by the high stimulus contrasts and small phase offsets used by Huang et al. [5]. Furthermore, strong neurophysiological evidence of substantial modulations in neural activity as a function of interocular phase difference [8]–[13] lead us to expect some phase effects at a perceptual level.

To address these concerns, we performed a binocular contrast matching experiment using horizontal sine-wave gratings over a wide range of contrasts (2–32%) and interocular phase differences (vertical disparities, 0–180°). The ‘target’ stimulus had a fixed contrast, with the phase relationship between the eyes varied experimentally. Observers compared the target to a ‘match’ stimulus, which had the same phase in each eye, but its contrast was determined by the observer's responses (see Materials and Methods). The results revealed a complex pattern of interactions between phase and contrast that was consistent across observers. Clearly then, models of binocular contrast perception must incorporate interocular phase.

## Results

**Figure 1:**
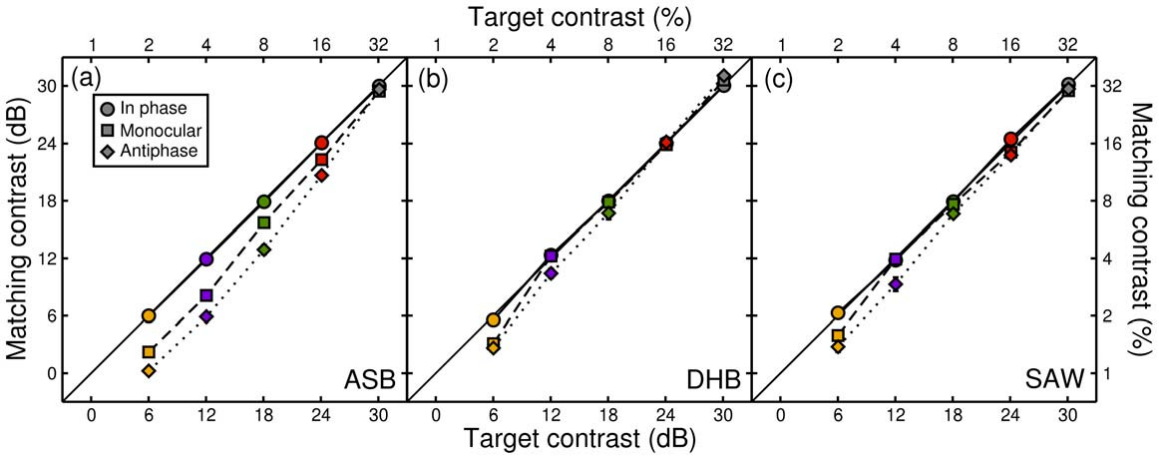
Perceived contrast for in phase, monocular and antiphase gratings. Symbol shape indicates target phase and binocularity, with colours representing target contrast. Each point is the mean of four PSE estimates. Error bars (±1SE across repetitions) are plotted, but in all cases are smaller than the symbols.

We first demonstrate that our matching method is reliable by plotting perceived contrast for the condition in which target phase is equal across the eyes (circles in Figure 1). In this condition, target and match had the same phase, and differed only in contrast (the match contrast being determined by the staircase). It is clear that observers were able to make veridical contrast judgements with high accuracy, with all contrast matches (circular symbols) lying very close to the line of unity. Figure 1 also shows the results of a monocular condition (squares), in which the target was shown to only one eye (the other eye saw mean luminance). At low contrasts, monocular targets appeared fainter than the binocular match, as the points lie below the veridical line, particularly for observer ASB (Figure 1a). At higher contrasts, monocular and binocular stimuli appear equal in contrast, consistent with previous work [14], [15]. A similar pattern is evident for the antiphase condition (diamonds), which in general had lower perceived contrast than either the in phase or monocular conditions. Note that at the highest target contrast (32%), all three conditions are perceived as equal in contrast (grey symbols).

**Figure 2:**
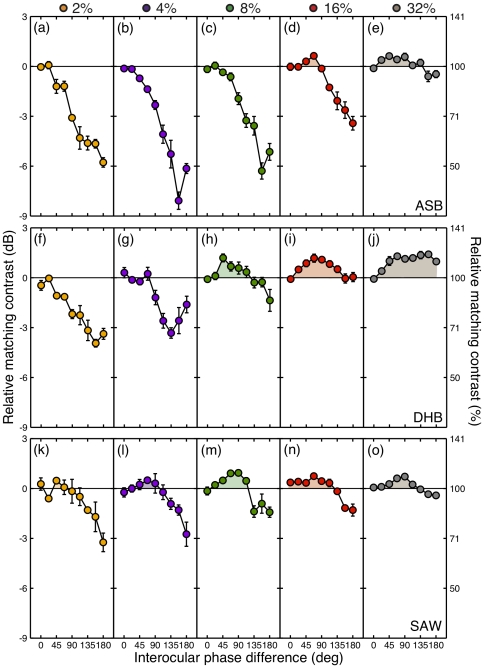
Perceived contrast as a function of interocular phase difference. Rows correspond to different observers, and columns (and colours) represent different target contrasts, given at the top of the figure. The data are plotted as matching contrasts relative to the physical target contrast, where a veridical match is 100% (or 0 dB). Error bars indicate ±1SE across four repetitions. The horizontal lines in each panel represent the null hypothesis, that there is no effect of interocular phase on perceived contrast.

Perceived contrast as a function of interocular phase difference is shown in Figure 2 for three observers (rows) at five target contrast levels (columns). The data have been normalized to their respective physical contrasts, such that for veridical perception (target = match) data would lie on the horizontal lines at 0 dB. It is immediately apparent that perceived contrast is not veridical across interocular phase differences, nor at all contrasts. At low contrasts (2%, 4%), perceived contrast is attenuated as the phase difference approaches 180° (Figure 2 panels a,b,f,g,k,l). This contrast reduction exceeds 6 dB (a factor of 2) for observer ASB, and reaches 3 dB (a factor of 1.4) for the other observers. At higher contrasts, there is an amplification of perceived contrast at intermediate phase offsets (∼90°) of 1–2 dB (shaded regions). This pattern is clear for all observers, though the transition from attenuation to amplification occurs at different interocular phase differences and contrasts for each observer.

We also performed two-way ANOVAs, with target contrast and phase difference as factors, and individual repetitions (n = 4) providing the multiple observations for each observer. There was a significant effect of phase for all observers (all *F*_8,135_>19, all *p*<0.001). Contrast effects were also significant (all *F*_4,135_>8026, all *p*<0.001), and remained so (all *F*_4,135_>10, all *p*<0.001) even when the data were normalized to the appropriate target contrasts (e.g. Figure 2). Interactions between the two variables were also significant (all *F*_32,135_>1.6, all *p*<0.05).

**Figure 3:**
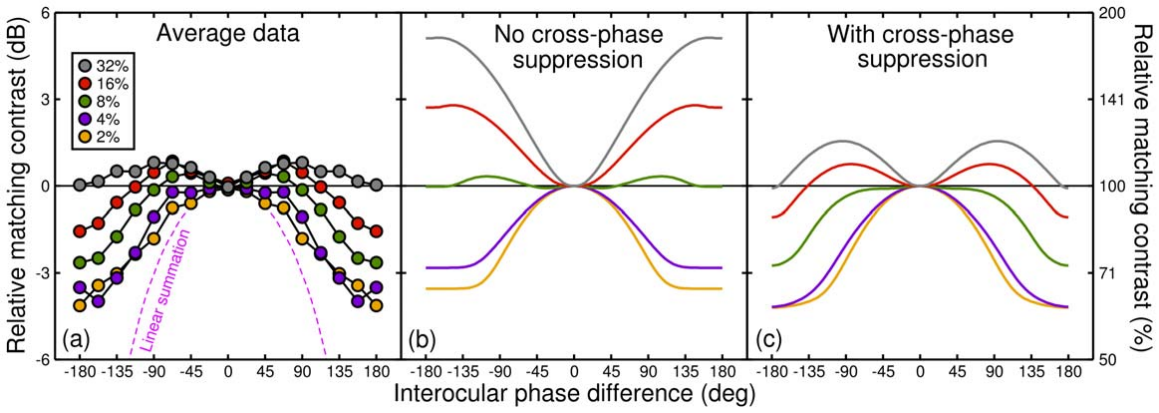
Average results and model predictions. (a) Data averaged across three observers (symbols), and the prediction for linear binocular summation (dashed pink curve). The data were normalized to the target contrast for each condition, and had a mean standard error across observers of 0.62 dB. (b) Predictions of a gain control model with no cross-phase suppression. (c) Predictions of the model with suppression between phase channels and *w* = 0.33. See text for model details.

As the individual results were qualitatively similar, we averaged them as shown in Figure 3a. The data are mirrored about 0° to remind the reader that both directions of phase offset were used in the experiment. The averaged data reveal a complex pattern of phase-dependent attenuation at low contrasts ceding to amplification at higher contrasts. To understand how this pattern arises we present a simple computational model, described in the following section.

### Models

To inform the development of a computational model, it is instructive to first explore the predictions of linear (binocular) summation of the two stimulus waveforms. This is shown by the pink curve in Figure 3a, and clearly underestimates the perceived target contrast over much of the range of phase differences. In particular, perceived contrast is zero when the stimuli are in antiphase because, being of equal contrast, they cancel entirely. This is clearly inconsistent with the experimental results, for which attenuation is never greater than 4 dB (i.e. a factor of about 0.63). For the situation where left and right eye contrasts are matched (as here), Ding & Sperling's [3] model produces identical predictions to linear summation, because the weight terms are balanced. The prediction of an elaborated version of that model [5], in which perceived contrast is entirely independent of interocular phase difference, fares even worse, with perceived contrast given by the horizontal line in each panel of Figure 3 (0 dB).

Clearly then, a successful model must solve two problems. First, the stimulus must be visible when in antiphase, which rules out direct summation of light and dark bars. Second, there should be differences between the functions for different matching contrasts. To achieve these aims, we constructed a model that was a variant of the two-stage binocular contrast gain control model of Meese, Georgeson and Baker [15], and subsequent related models [2], [16], [17]. The original model [15] used peak Michelson contrast (a single number) as its input to each eye, and made no predictions regarding phase. Later versions [16], [17] were extended to include area summation mechanisms and phase-specific filtering, and used full 2D images as inputs. The present model is a 1D simplification of these models, that processes the full spatial waveform (e.g. a sinusoid) of the target in each eye. The sequence of model stages was as follows:

- The stimulus waveforms in each eye were half-wave rectified in separate, positive and negative phase channels (positive channels respond to bright bars, negative channels to dark bars)
- The rectified waveforms passed through a gain control which included suppression from the other eye (see equations 3 and 7 below)
- Linear binocular summation of these responses across the two eyes
- A second gain control stage, followed by pooling over phase and space

The input waveforms for the left eye were sinusoidal functions of luminance, and after rectification were described in the positive channel (superscript *+*) by,
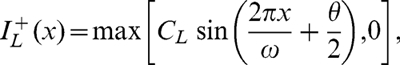
(1)and in the negative channel (superscript *−*) by,
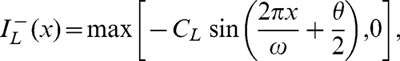
(2)with equivalent expressions for the right eye (*I_R_*). *C_L_* represents left eye Michelson contrast (in percent), *x* is spatial position (in pixels), *ω* is the spatial period (in pixels per grating cycle) and *θ* is the interocular phase difference (in radians). [Note: we could have included a pair of identical linear spatial filters (receptive fields) to capture the stimulus in each eye, but since *any* such filters would not alter the sinusoidal form of the input, and would not alter the relative amplitude nor the relative phase between the eyes, this initial filtering can be safely omitted without loss of generality.]

The early gain control equation was identical to the first stage of the Meese et al. [15] model, and was defined as
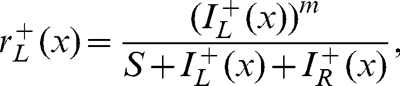
(3)where the parameters were either based on previous work (*m* = 1.3) or adjusted by hand to produce appropriate behaviour (*S* = 6). There was an equivalent expression for the right eye (*r_R_^+^*) and for the negative phase channels (*r_L_^−^, r_R_^−^)*. The second gain control stage, which includes binocular combination, was given by,
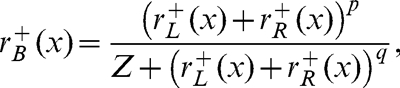
(4)with the parameter values (*Z* = 0.1, *p* = 8, *q* = 6) similar to those from previous work [15]. These responses were then pooled over phase,
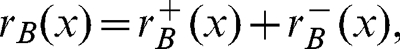
(5)producing an output analogous to a complex cell in V1. Finally, responses were pooled over space,
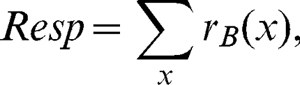
(6)to give a single output which was used to predict perceived contrast. Note that because summation was linear in equations 5 and 6, the order of these stages is arbitrary.

This model represents a binocular mechanism tuned to zero disparity. A perceptual match was deemed to occur when *Resp* was equal for the target and matching waveforms. Note that the positive and negative phase channels give equal responses to sinewave stimuli. This means that summing across the two phases (equation 5) makes no difference to the model behaviour, as it merely doubles the value of *Resp* in all conditions. However, we include both phase terms here for generality. A recent 3-stage model, constructed to account for detection [16] and discrimination [17] of contrast across space and eyes featured a further gain control stage following spatial pooling. However, this is irrelevant for contrast matching and was omitted for parsimony.

The model predictions for the present experiment are shown in Figure 3b. They meet the two main aims outlined above: perceived contrast is nonzero for antiphase gratings, and there are large differences between the different contrast conditions. However, the character of the curves is wrong for the higher contrast levels (grey and red curves). This problem can be identified with the interocular suppression term in equation 3, which falls to zero for antiphase stimuli (because the rectification removes the troughs in one eye which line up with the peaks in the other). To compensate for this, we introduced an interocular cross-phase suppressive term [2], [17], so that equation 3 becomes:
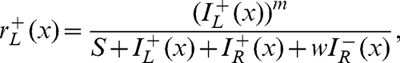
(7)where *w* is a weight parameter, and the other terms are as described previously. (Note that a within-eye cross-phase term (*I_L_* for the above equation) has no effect in our contrast matching experiments and was omitted for simplicity.) With this simple refinement and an intermediate weight parameter of *w* = 0.33, the model's behaviour improves markedly to that shown in Figure 3c. (Note that Figure 3b is equivalent to setting *w* = 0, and that at very large values of *w* (not shown) the model's behavior approaches that for linear summation (pink curves in Figure 3a), for all contrasts.) Our preferred model has 6 parameters, of which four (*m, p, q* and *Z*) were based on previous work, and two (*S, w*) were adjusted to produce appropriate behaviour for the present results.

The model captures the transition from phase-disparity dependent attenuation to amplification as target contrast increases. This is an emergent property of the early gain control architecture, where the parameter *S* dominates the denominator of equation 3 (and equation 7) at low contrasts, but not at high contrasts, resulting in a change in model behaviour. Individual variations in the value of *S*, *w*, or any of the other parameters might explain the small observer differences apparent in Figure 2. Our aim was to present a model along similar lines to previous variants [15]–[17] to illustrate plausible operations behind the phase dependencies of binocular combination of gratings, rather than to optimize the parameters by fitting to the data.

## Discussion

We report the results of a binocular contrast matching experiment in which interocular phase difference and target contrast were manipulated. We find evidence for changes in perceived contrast that are dependent on both variables. The pattern of interactions can be modeled by assuming interocular suppression within and between polarity-specific (light or dark) channels, followed by pooling over eyes and space within each polarity-specific mechanism.

One obvious question to ask is why we find that interocular phase difference affects perceived contrast, whereas Huang et al. [5] did not. The primary reason for this is that the range of conditions investigated by Huang et al. falls in the region of stimulus space in which the least variation occurs (e.g. red and grey symbols in Figure 2 between 0 and 90°). The present result is therefore not a failure to replicate their findings, but an extension of their approach that has important consequences for their conclusions. However, there are several methodological differences between the two studies that might also contribute to the different conclusions. First, the stimulus duration used by Huang et al. was unlimited in principle, and in practice probably in the order of several seconds. Ding & Sperling [4] demonstrated that perceived phase varies as a function of presentation duration, and the same could well be true of perceived contrast. A long stimulus duration might promote binocular rivalry alternations, perhaps meaning that only one eye's image was seen, and that was at its veridical contrast. A second important difference concerns bias from the use of the method of adjustment in the Huang et al. study. Since observers knew which side of the stimulus was the target and which was the match, this knowledge might have influenced their contrast judgements, perhaps through the use of an implicit standard. Our 2IFC paradigm used brief, central presentations (with random ordering of target and match) thereby avoiding these shortcomings.

As a further methodological point, we avoided using vertical stimuli here because we did not want horizontal vergence movements to influence our results by negating or reducing a phase offset. For vertical stimuli, it is also likely that neural channels sensitive to binocular disparity might complicate the results, since stimuli with an appropriate phase-disparity are optimal for such channels. We acknowledge that disparity channels that are sensitive to the vertical disparities in our stimuli might exist (see [18]), but note that they would only be expected to reduce the magnitude of any phase effects on perceived contrast. In sum, our findings provide a conservative estimate of how activity in a single (zero-disparity) channel is influenced by interocular phase difference. Furthermore, our model did not require nonzero-disparity channels to account for the results (i.e. there was no binocular combination across mechanisms tuned to different phases, only within a mechanism of a particular phase or polarity).

To offer some insights into why the model behaves as it does, we make a few observations. First, at low contrasts, the attenuation at large phase differences occurs because there is no binocular summation in these conditions (consistent with results at detection threshold [6]). This difference reduces at higher contrasts, as the saturation constant (*S*) contributes proportionally less to the denominator of the early gain control, and the suppressive terms contribute proportionally more. The dominance of suppressive terms on the denominator results in the property of ‘ocularity invariance’, whereby high contrast monocular and binocular stimuli appear equal in contrast (grey symbols in Figure 1; and [14]). This happens because,
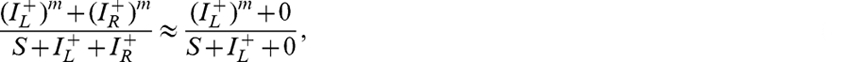
(8)in conditions where *S* is small compared with *I* (the antiphase suppressive terms, 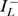 and 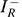, are zero here for the case of in-phase stimuli).

Second, at high contrasts the ‘bumps’ in the model predictions are caused by changes in the effective level of interocular suppression with phase difference. As the interocular phase difference increases there is less pointwise correspondence between excitation and same-polarity interocular suppression, so the model response (and hence perceived contrast) increase (grey and red curves, Fig. 3b). At greater phase differences the opposite-polarity suppressive terms also begin to contribute meaningfully, and this additional suppression reduces perceived contrast (grey and red curves dip back down again in Fig. 3c).

### Conclusions

The results of a contrast matching experiment demonstrate that perceived contrast does depend on interocular phase difference. This shows that the conclusions of Huang et al. [5] do not generalise to lower contrasts and larger interocular phase differences than were tested in their study. Unlike in their model, perceived contrast was not invariant with phase disparity. The observed pattern of contrast attenuation and contrast amplification can be explained by a simple model involving binocular summation and interocular suppression. Developing computational models of perception under conditions of binocular phase offset might be important in clinical settings (e.g. amblyopia [19], [20]) and for predicting subjective responses to 3D display and cinema technologies.

## Materials and Methods

### Ethics Statement

All participants gave written informed consent, and procedures were approved by the Aston University Ethics Committee.

### Apparatus & stimuli

All stimuli were presented on a Clinton Monoray monitor (Cambridge Research Systems Ltd., Kent, UK) running at a frame-rate of 120 Hz, driven by a ViSaGe stimulus generator (CRS Ltd) controlled by a PC. We used ferro-electric shutter goggles (CRS, model FE-1) and a frame interleaving technique to enable dichoptic presentation with negligible crosstalk. The goggles attenuated the mean luminance of the display to 10 cd/m^2^.

Stimuli were horizontal 1 c/deg sinusoidal gratings, windowed by a raised cosine envelope, with a full width at half height of 4° and a total extent of 5°. We chose to use these stimuli instead of those used by Huang et al. [5] for consistency with our previous and current work on binocular combination (e.g. [2], [14], [15]). Unlike Huang et al. [5] we did not require phase matching judgements at the same time as the contrast-matching.

### Procedure

The observers viewed the display through the goggles, which were mounted on a chin rest 1 metre from the display. We used a two-interval contrast matching paradigm to measure perceived contrast. The target stimulus had a fixed Michelson contrast of 2, 4, 8, 16 or 32%, and a phase difference (vertical disparity) between the eyes of θ°, where θ was drawn from the range 0–180°. The left and right eye targets were offset by ±0.5*θ°, so that their average phase was always equal to that of the matching stimulus. The matching stimulus was binocularly in-phase, and had either a peak or a trough aligned with a central fixation point. The matching stimulus had a variable contrast that was controlled by pair of 1-up-1-down staircases. For one staircase the positive target phase increment was shown to the left eye, for the other staircase it was shown to the right eye. We also included a monocular condition, in which the target was shown only to one eye (though the match stimulus was still binocular).

The observer's task was to judge which of the stimuli from two temporal intervals (each 200 ms in duration, separated by 500 ms) appeared higher in contrast. One interval contained the target, and the other contained the match, presented in random order, each indicated by a beep. Responses were made via a two-button mouse, and no feedback was given for this subjective task. Conditions were blocked by target phase offset and contrast, and observers completed four repetitions of each condition, where each repetition was a randomised order of blocks. We fitted the resulting psychometric functions using a cumulative log-Gaussian to estimate the point of subjective equality (PSE), at which the target and match appeared equal in contrast.

### Observers

Three psychophysically experienced observers completed the experiment. Two were authors (DHB, SAW) and the third was a postgraduate student (ASB) who was not aware of the aims of the experiment. Observers were optically corrected if required and had good stereoacuity and no known anomalies of binocular vision.

## References

[1] Levelt WJM (1966). The Alternation Process In Binocular Rivalry.. Br J Psychol.

[2] Baker DH, Meese TS (2007). Binocular contrast interactions: dichoptic masking is not a single process.. Vision Res.

[3] Ding J, Sperling G (2006). A gain-control theory of binocular combination.. Proc Natl Acad Sci U S A.

[4] Ding J, Sperling G, Harris L, Jenkin M (2007). Binocular combination: measurements and a model.. Computational vision in neural and machine systems.

[5] Huang CB, Zhou J, Zhou Y, Lu ZL (2010). Contrast and phase combination in binocular vision.. PLoS One.

[6] Simmons DR (2005). The binocular combination of chromatic contrast.. Perception.

[7] Rose D, Blake R, Halpern DL (1988). Disparity range for binocular summation.. Invest Ophthalmol Vis Sci.

[8] Chino YM, Smith EL, Yoshida K, Cheng H, Hamamoto J (1994). Binocular interactions in striate cortical neurons of cats reared with discordant visual inputs.. J Neurosci.

[9] Ohzawa I, Freeman RD (1994). Monocular and binocular mechanisms of contrast gain control.. Computational vision based on neurobiology.

[10] Sengpiel F, Blakemore C, Harrad R (1995). Interocular suppression in the primary visual cortex: a possible neural basis of binocular rivalry.. Vision Res.

[11] Sengpiel F, Jirmann K-U, Vorobyov V, Eysel UT (2006). Strabismic suppression is mediated by inhibitory interactions in the primary visual cortex.. Cereb Cortex.

[12] Smith, 3rd EL, Chino YM, Ni J, Cheng H, Crawford ML (1997). Residual binocular interactions in the striate cortex of monkeys reared with abnormal binocular vision.. J Neurophysiol.

[13] Smith EL, Chino YM, Ni J, Ridder WH, Crawford ML (1997). Binocular spatial phase tuning characteristics of neurons in the macaque striate cortex.. J Neurophysiol.

[14] Baker DH, Meese TS, Georgeson MA (2007). Binocular interaction: contrast matching and contrast discrimination are predicted by the same model.. Spatial Vision.

[15] Meese TS, Georgeson MA, Baker DH (2006). Binocular contrast vision at and above threshold.. J Vis.

[16] Meese TS, Summers RJ (2009). Neuronal convergence in early contrast vision: binocular summation is followed by response nonlinearity and area summation.. J Vis.

[17] Meese TS, Baker DH (2011). Contrast summation across eyes and space is revealed along the entire dipper function by a “Swiss cheese” stimulus.. J Vis.

[18] Cumming BG (2002). An unexpected specialization for horizontal disparity in primate primary visual cortex.. Nature.

[19] Baker DH, Meese TS, Hess RF (2008). Contrast masking in strabismic amblyopia: attenuation, noise, interocular suppression and binocular summation.. Vision Res.

[20] Huang CB, Zhou J, Lu ZL, Zhou Y (2011). Deficient binocular combination reveals mechanisms of anisometropic amblyopia: signal attenuation and interocular inhibition.. J Vis.

